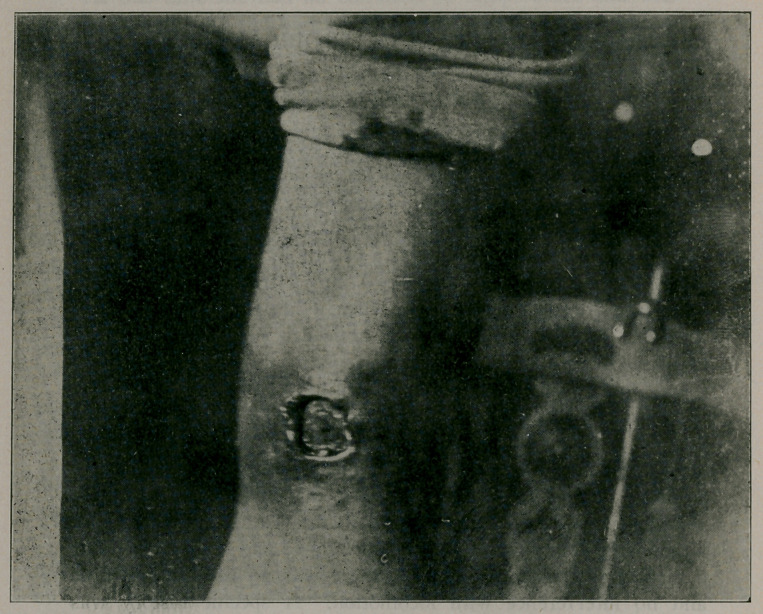# Report of a Case of Tropical Ulcer

**Published:** 1908-10

**Authors:** W. E. Wilmerding

**Affiliations:** Atlanta, Ga.; Candler Building


### REPORT OF A CASE OF TROPICAL ULCER.

RY DR. W. E. WILMERDING, ATLANTA, GA.

Mr. W., aet. 32; father living, aet. 58 in good health; mother died at 40 of pulmonary tuberculosis, as did both of her parents. Always had good health until December, 1905, when a wound made by a sharpened bamboo just above right ankle, while in the Philippine Islands, serving as a private in the U. S. Army, became infected and later developed into a tropical ulcer. This ulcer lasted until August, 1906, when it healed and he returned to duty. In November, 1906, he noticed a small swelling, hard and brawny and about the size of a pecan, in the right popliteal space; this gradually increased in size until January, 1908, when it was about three inches in diameter, nearly round, very hard and of a dark red color. Late in January he noted a fissure across the center which presented the appearance of an incised wound. At this time he came under my observation, and in a few days this fissure had enlarged to the size of a silver dollar, was nearly round, had overlapping edges, and had for a floor a dirty grey tenacious slough. There was no pain at any time and no interference with locomotion. A specific history was denied, and there was no evidence of any specific lesion elsewhere on the body. Under the use of pure carbolic acid applied every other day to this slough and packing the ulcer with iodoform gauze, and a thorough irrigation with a 1 to 500 permanganate solution, this slough disappeared and the ulcer presented a clean cut border and an uneven floor and a depth of about 1 1-2 inches. Granulation gradually filled up this cavity and after several months only a slight scar was left. The case was dismissed May 11th, 1908 as cured, but the patient returned in a few weeks with a small hard nodule in the calf of the same leg, which presented every indication of being the beginning of another tropical ulcer. Incision and complete removal of the mass was urged, but refused, and after a few weeks this mass showed a softened area which when opened liberated a small amount of thin pus and disclosed a dirty grey slough which extended under the edges of the wound for some distance. Similar treatment was pursued as with the first ulcer and the slough had disappeared and granulation had set in when the patient went to Camp Taft. During his absence of ten days the legging on his right leg made several abrasions around the ulcer, all of which became infected and small superficial ulcers formed, which yielded readily to treatment, and the case was dismissed September 14th, 1908.

A section of the periphery of the first ulcer was examined by Dr. H. F. Harris as were several dressings, but no special organism was found.

Candler Building.

## Figures and Tables

**Figure f1:**